# Managers’ Use of Organizational Resources when Supporting Employees with Common Mental Disorders: A Swedish Qualitative Study

**DOI:** 10.1007/s10926-025-10293-4

**Published:** 2025-04-19

**Authors:** Ellinor Tengelin, Lisa Björk, Linda Corin, Gunnel Hensing, Carin Staland-Nyman, Christian Ståhl, Monica Bertilsson

**Affiliations:** 1 Department for Health Sciences, Mid-Sweden University, Rehabilitation Science, 831 25 Östersund, Sweden; 2 Institute of Stress Medicine, Region Västra Götaland, 462 80 Gothenburg, Sweden; 3 Department of Work Science and Sociology, University of Gothenburg, 405 30 Gothenburg, Sweden; 4 Insurance Medicine, School of Public Health and Community Medicine, Institute of Medicine, University of Gothenburg, 405 30 Gothenburg, Sweden; 5 School of Health and Welfare, Halmstad University, 301 18 Halmstad, Sweden; 6 Division of Education and Sociology, Department of Behavioural Sciences and Learning, Linköping University, 581 83 Linköping, Sweden

**Keywords:** Common mental disorders, Managers, Occupational health, Organizational resources, Qualitative research

## Abstract

**Purpose:**

Among the diverse tasks of managers, handling employees with common mental disorders (CMDs) is perceived as particularly challenging. Little is known about the organizational resources that managers use in these situations. The aim of this study was to explore the organizational resources that managers use when handling employees with CMDs and how they experience their use.

**Methods:**

An interview study inspired by the critical incident technique was designed. Swedish managers (n = 35) were recruited if they were managers in a private company with at least 50 employees and had experience supporting one or more employees with a CMD in the last 2 years. An incident was defined as a situation when a manager needed to support an employee with a CMD.

**Results:**

The analysis revealed managers’ use of seven kinds of resources, and how the resource helped or hindered support to employees: (1) routines and structures; (2) frames for action; (3) training and education; (4) expert functions; (5) safety representatives from worker unions; (6) social support from colleagues and management; (7) interaction with employees. Secondary findings were personal and external resources, widening the study’s organizational focus.

**Conclusions:**

This study is one of the first to explore managers’ real-life experiences of the support available in their organization when managing employees with CMDs. Managers’ experiences may help organizations provide structures for the management of individual cases of CMD at work. Resources flexible to the needs of managers in specific situations were experienced as supportive by participants in this study.

## Introduction

Responsibilities regarding health, safety and rehabilitation of employees are executed by managers. Managers’ room for action when it comes to fulfilling these responsibilities is largely determined by the organizational context and its preconditions [1–3], which influences and enables organizational behaviour [4]. To the best of our knowledge, there is limited knowledge on how managers experience organizational preconditions and if and how these preconditions facilitate or hinder the daily handling of employees with common mental disorders (CMDs).

CMDs are described as challenging in working life [5]. Although a common reason for sickness absence, managers claim that they seldom meet employees with CMDs at the workplace level. Only two-thirds of participating managers in a Swedish study (n = 3032) had encountered no or only one employee with a CMD in the last 2 years [6]. This might be due to a lack of employee disclosure and associated stigma [7]. However, many managers are likely to encounter employees with CMDs because the OECD estimates that 15% of the working force experiences mental health disorders, and once sick-listed, many tend to be on long-term sick-leave [5]. In some situations, managers may find it challenging to handle individuals with CMDs [1, 8–10]. Only 25% of the managers in the Swedish project study reported feeling confident about supporting an employee with a CMD [11]. Porter et al. [12] found that managers struggle to comprehend what CMDs are and that they lack established resources to support employees with mental health problems. Further, managers feel uncertain and that they are left alone with the responsibility for an employee’s rehabilitation process [13]. Quinane et al. [14] described managing employees with mental health problems as filled with tensions; for example, struggling with whether employees’ mental health problems should be viewed as their own private matter or an organizational responsibility [14]. Martin et al. [1] interviewed Australian managers (n = 24) who found it difficult to handle their own workload, keep a professional distance and manage the emotional impact on their personal lives while supporting an employee with a CMD.

Preconditions that enhance managers’ agency are important for their ability to support employees [15]. Organizational context shape, constrain, and enable leadership behavior [4, 16–18], but to take contextual factors into consideration is still rare in research on managers’ responses to mental health issues in the workplace [19–22]. The framework for this study is based on an understanding of organizational context as situational opportunities and constraints that affect the occurrence and meaning of organizational behaviour [4]. Gary Johns distinguishes different levels of context and features that influence behaviour and attitudes in organizations. At the discrete level we find phenomena such as work design and social cohesion [4, 18], at the meso level industry characteristics and organizational demography play a crucial role, while factors related to national culture or macro-economical conditions exist at the omnibus level [23]. Johns’ framework provides an overview of the different levels of organizational context that impact leadership behaviour. In the present study, we acknowledge this multilayered context and suggest that some of organizational factors might be considered to be organizational resources in the sense that they facilitate for managers in taking on the challenging task of handling employees with CMDs. What these organizational resources are and how managers experience them is thus a matter for empirical investigation.

In previous research on managers’ preconditions, Biron et al. [3] studied the psychosocial safety climate in a 3-month follow-up of an organizational implementation, and psychosocial work factors were seen as resources affected by the wider organizational context, determining the quality of managerial practices. Resources facilitating managers’ work were suggested to be an organizational climate that included the commitment of senior management, the management of mental health as a priority, organizational communication of psychosocial health and safety, and the degree of managerial and other stakeholder involvement [3].

Mental health stigma adds to the organizational climate and the complexity of handling CMDs in organizations [24]. In the workplace, colleagues may hesitate to work alongside people with mental health problems [25, 26]. Employees might avoid disclosing their CMD problems to managers because of stigma [27, 28], and this makes it difficult for managers to detect the problem [29] and to approach the subject among their employees [1]. A common dilemma is how managers balance confidentiality and employee privacy and colleagues’ need for information [1, 10, 29]. Furthermore, at the managerial level, managers in the organization might have negative attitudes to CMDs [30, 31]. On a structural level, cultural norms, workplace policies, and routines are either supportive or hinder managers’ efforts to support these employees [32]. A recent study showed that contextual stigma at different levels (among employees, managerial colleagues, and senior management) was negatively related to managers’ actions to prevent sickness absence of employees with CMDs [33].

Managers need adequate resources to be able to handle CMDs in the workplace [34]. In a mixed-method study of managers in Australia, Johnston et al. [10] identified that managers need knowledge of systems, processes and procedures to handle employees with health problems. However, these preconditions imply that supportive systems and procedures are in place in companies. Bastien and Corbière [35] identified factors important for work accommodations of which one was “to equip and support the manager”, however, they found only 3% had put that action in place. In workplaces where knowledge about CMDs is extensive, managers and employees are more likely to discuss questions about CMDs [36]. In cross-sectional studies, we found associations between working in organizations that address mental health issues (e.g. through lectures to employees) and managers’ self-reported prevention of CMDs and their use of work accommodations for employees with CMDs [37, 38]. Guidelines on how to act if an employee has mental health issues were a useful resource for managers [39], and organizational culture, policies and practices are potentially important for managerial support for employees with mental health problems [21]. In summary, CMDs are a complex situation in workplaces and may be challenging to handle for managers, depending on the severity and situation. Studies suggest that contextual factors may play a role in the quality of managers’ support [40].

Knowledge is lacking on the organizational resources that managers use to handle employees with CMDs and how they experience their use. This knowledge could provide valuable insight into how employers can better facilitate managers in their responsibility to prevent ill-health and support rehabilitation among employees with CMDs. The aim of this study was to explore the organizational resources that managers use when handling employees with CMDs and how they experience their use.

## Methods

### The Critical Incident Technique

**Fig. 1:**
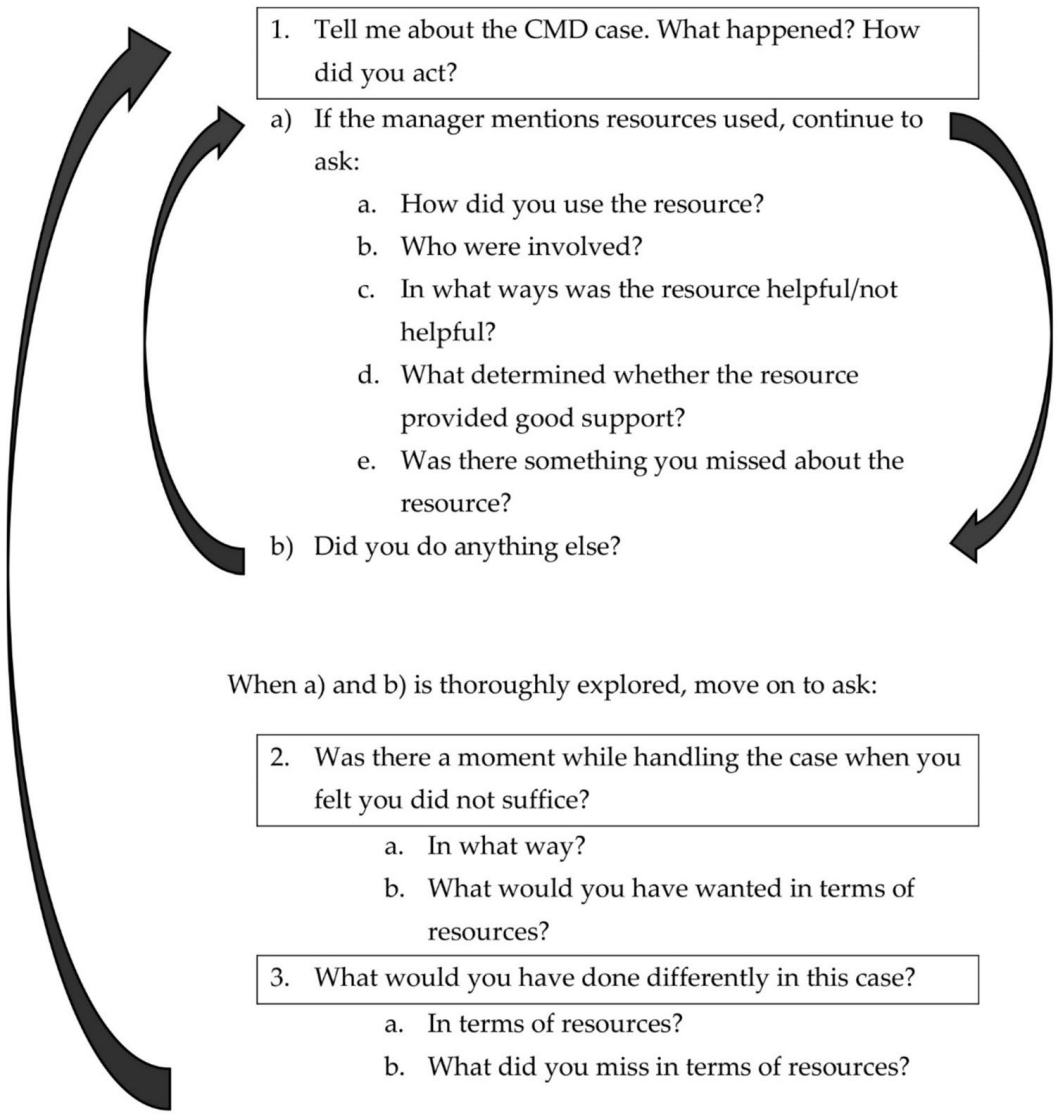
Interview guide. When question 3 was finalized, the interview continued with the next case and started with question 1 again

The critical incidence technique (CIT) was chosen as a method to generate and analyse data because of its aim to examine behaviour and actions that are relevant to understanding a specific work situation [41]. In the present study, we defined a situation or critical incident as follows: when a manager needs to support an employee with a CMD. The technique allows concrete situations in an employee case to be recalled in detail, which helps us get a realistic description of managers’ experiences of the use of resources (Fig. 1). Further, the method is suitable for analysing work situations that have”a flexible or undefinable number of correct ways to behave” [42]. Because of its attention to actions in a specific situation, findings from a CIT study can bring understanding into what works efficiently in a situation and what does not and can therefore be used to help organizations develop interventions to overcome barriers that hinder certain behaviours [43], e.g. managerial support in situations where employees have CMDs.

## Setting

The study was carried out among Swedish managers within the private sector. Employers in Sweden are obliged to provide managers and team leaders with the knowledge and resources needed for a safe organizational and social work environment, including prevention and management of detrimental workloads [44]. Further, the employer is obliged to organize work so that the statutes of the Swedish Work Environment Authority can be practically applied by managers. For example, managers themselves need a reasonable workload, support and sufficient mandate to be able to take on their responsibilities.

The Swedish labour market is characterized by collaboration between employers and unions. Employers with at least five employees are obliged to have a safety representative at their workplace [45]. Safety representatives are usually members of a union organization, and their primary function is to ensure that the work environment adheres to the relevant laws and regulations.

Sickness insurance in Sweden is universal, covering all citizens from the age of 18 years. It regulates employer responsibility for the rehabilitation of sick-listed employees back to work. The first 7 days of sick-leave are self-certified in the Swedish sickness insurance scheme. A sickness certificate by a physician is required from the eighth day. Employees can be on sick-leave for 25%, 50%, 75% or 100% of working time.

Further, the occupational health service (OHS) is an independent external expert service that provides support for workplace environment and rehabilitation issues. Swedish companies may choose to engage and fund the OHS when their internal expertise is insufficient for systematically managing work environment challenges [44]. Forty percent of employees in Sweden lack access to OHS and 50% of these work in companies with less than 50 employees [46]. The main tasks for the OHS include rehabilitation and measures to prevent sickness absence as well as decreased work performance.

## Participants and Procedure

In qualitative research, participants should represent a variety of experiences with the topic under study. Inclusion criteria in this study were being a manager in a private company with at least 50 employees and having experience supporting one or more employees with CMDs in the last 2 years. We recruited participants in two ways: [1] by contacting managers directly through managers’ union organizations and [2] by contacting companies and asking them to inform managers in their organization about the study. During spring 2022, information about the study was distributed through two union organizations for managers in Sweden; four managers were recruited this way. During the same period, the author group contacted companies in the service sector (pink-collar jobs, referring to female dominated occupations in care and service work), the manual labour sector (blue-collar jobs), and the office sector (white-collar jobs) with information about the study; 27 managers were recruited this way. In addition, a business developer organization informed companies about the study; one manager was subsequently recruited.

In a CIT design, the number of incidents determines when the sample is complete [38]. After interviewing managers during spring 2022, we had a low number of incidents from managers in the pink sector. Therefore, in August 2022, we contacted companies in the service sector to recruit additional managers, and another three managers were recruited this way. In total 35 managers were recruited; their demographics are shown in Table 1. Another five managers were initially scheduled for an interview but were excluded because they did not fulfil the inclusion criteria or withdrew without giving any explanation.

**Table 1:** _Participants’ demographics (n = 35)_

Characteristics	Number
Gender	
Female	14
Male	20
Non-binary	1
Age (years)	
Range	27–60
Mean	42
Level of education	
Secondary education	13
Degree from college/university	18
Other post-secondary education	4
Industry	
Blue collar	16
White collar	13
Pink collar	6
Company size	
Small- and middle-sized companies (50–250^a^ employees)	9
Large (251–1000 employees)	6
Very large (≥ 1001 employees)	20
Managerial position	
Senior manager, CEO	1
Middle manager	14^b^
First-line manager	16
Supervisors	3
Expert manager	1
Managerial experience (years)	
Range	10 months to 25 years
Mean	9.1
White collar	
Range	10 months to 17 years
Mean	9.9
Blue collar	
Range	1–16
Mean	7.1
Pink collar	
Range	5.5–25
Mean	12.7
Span of control per industry	
White collar	
	6
11–30	6
31–50	1
Blue collar	
	6
11–20	10
Pink collar	
19–30	3
31–53	3

^a^One company had fewer than 50 employees but belonged to a corporate group where organizational resources could be accessed

^b^Three of the middle managers were also first-line managers (for different employee groups)

## Data Collection

In CIT, data on behaviours are collected from retrospective self-reports during interviews [41] to understand participants’ subjective world [47]. An interview guide was designed to capture managers’ descriptions of actions in situations where they supported an employee with a CMD. Questions targeted what they did, what resources they sought and used, and whether and how the resources were useful. The intent was to receive rich descriptions of how the resources were used by managers.

Pilot interviews were performed in December 2021, one each by ET (first author) and MB (last author). In subsequent discussions with the author group, slight alterations were made to the interview guide to better focus on how resources could help the manager. Interviews were then carried out from February to October 2022; ET interviewed 22 participants and MB interviewed 13. When the interviewers had each completed two interviews (by March 2022), both interviewers listened to the four interviews and then critically discussed the quality of the interviewing from a CIT perspective. Calibrations on the wording and structure of the interview guide were made to more clearly target the resources used by the managers and to ensure the interviews were carried out similarly by both interviewers. The interview guide was re-structured into a flowchart to facilitate repetition of questions that deepened participants’ descriptions of resources and how they were used (Fig. 1). The interviewers’ shared understanding of the study aims and interview guide was clarified in this way; this is particularly important when there is more than one interviewer.

Interviews were carried out digitally and lasted between 23 and 58 min with some time before and after the interview to introduce and finish off the session (not recorded). Interviews were recorded via video communication platforms and sound files were transcribed continuously by a professional transcribing firm. ET ensured the quality of the transcriptions by listening to sections of the sound file that the firm found unclear and clarifying the transcript.

## Data Analysis

The analysis phase started in autumn 2022 when all authors participated in identifying relevant data. ET read through all transcripts, identified managers’ use of resources, coded these, and sorted them into a matrix (showing what kind of resource was used in the incident and how the resource was used by the manager). Co-authors received the matrix and the transcripts to validate and add to ET’s identification.

The organizational resources were then analysed and described in more detail by carrying out a thematic analysis, resulting in seven themes. This method is recommended by CIT researchers [48] to create an understanding of the central patterns in qualitative data [49]. Within each theme, inductive categorizing incidents began. The aim of the study was used as a “frame of reference” [47] from which incidents were sorted and categorized. Data were coded into positive and negative experiences as well as requests for improvement, which allowed the nuances in experiences of each resource to be described. Even though the aim was to explore organizational resources, data showed that the managers used external resources, as well as personal and private resources. The use of resources was therefore grouped into three levels (external, organizational, and personal resources) widening the frame of reference. The author group continuously participated in discussions on the developing analysis.

## Results

Managers’ organizational preconditions to support employees with CMD were explored through managers’ use of organizational resources. Seven categories of organizational resources were identified (Table 2).

**Table 2:** _Overview of the findings pink_

Themes	Sub-themes
Routines and structures	
Frames for action	Sufficient economic preconditions
	Enough time in the workday
	Possibility for flexibility
Training and education	
Expert functions	HR department
	OHS supporting the employee
	OHS supporting the manager
Safety representative	
Social support from colleagues and management	The senior management team
	One’s own manager
	Managerial colleagues
	Team leaders
Interaction with employees	Social climate at the workplace
	An involved employee group
	Physical proximity to employees

HR, human resources department; OHS, occupational health service

## Routines and Structures

Managers experienced that organizational routines and structures could increase clarity in difficult situations and provide them with a sense of reassurance even though they felt insecure about how to handle issues of CMDs. Clear guidelines could create a shared language in the organization that facilitated and de-dramatized communication, and they further demarcated managers’ responsibilities and ensured that employees received the help they needed. Even though CMDs were still seen as a difficult issue, causing much worry for the employee as well as insecurity in how to act as manager, following firmly established routines could at least give managers a sense of security in that they had acted according to the rules. In a situation where an employee with a panic disorder had repeatedly been absent from work over a year, manager 10 was self-critical and expressed that he should have caught the employee’s mental struggles earlier. He recalled having used the company guidelines for dialogues around health issues, and even though it did not help him pick up on what was really bothering the employee, he felt that it was a source of relief:

It is helpful in a way that I know that I have done what I should. I feel like I have done what is expected of me. And it’s comforting knowing that.

However, without experience and knowledge of CMDs, it was described as challenging to translate written guidelines into hands-on advice. Occasionally, guidelines targeted the wrong level of the organization, describing the organization’s ideal process around employees with CMDs but not actually providing managers with practical guidance. Sometimes guidelines were written too generally and were not adapted to the specific conditions that faced managers. On the other hand, too detailed guidelines could be an obstacle to finding more flexible solutions for the employee. In a situation involving an ongoing case of depression, manager 33 was aware of having to continually be attentive to the employee and her changing life situation. Plans that were made in advance according to the company routines did not always work out:

You have to be really flexible I think, it doesn’t work just having a blueprint that shows how you should do it. When you worked halfway through it [the guideline], you realize that you have to start over. You have to be open to see when things don’t work out and we have to rethink.

## Frames for Action

Managers’ control and decision-making over their actions were restricted by three organizational resources.

### Sufficient Economic Preconditions

Having the possibility to purchase services, aids, or to hire substitutes was described as critical for managers in supporting the employee. Manager 22 thought the support from the OHS to the employee was insufficient and therefore requested another source of support. The company then paid for external coaching sessions for the employee, which the manager found to be very helpful; the coaching worked effectively and strengthened the employee:

Even though this of course is a very expensive service, there was no hesitancy on behalf of the company to approve this.

Managers had also experienced being denied economic resources and did not trust that their company would meet their needs. Manager 27 described a situation where support from an external OHS would have been useful:

I don’t have a lot of experience with [company name] yet. But my feeling, unfortunately, is that when it comes to those things, they cost money. […] If I were to say that I need support in this case, I’m not sure I should receive it.

### Enough Time in the Workday

Adequate time resources were a prerequisite for being able to support employees. Managers spent more time listening to and communicating with employees with CMDs than they did with other employees. Lack of time could force managers to overlook signs of employees not feeling well mentally, rather than immediately initiating a dialogue and documenting the process. When there was not enough time for every managerial task, either the company’s core business suffered or conversations with employees had to be deprioritized. In a situation where an employee was repeatedly absent from work for unclear reasons, manager 2 felt he was left to handle the case without adequate support:

It can be pretty stressful, and it takes a lot of time away from productivity, the core business, which you want to spend time on and develop and work strategically with.

### Possibility for Flexibility

It was critical to have the possibility to work flexibly, rather than being limited to standardized solutions in supporting employees. Managers were helped by company regulations that allowed flexibility on when and where work should be carried out, which allowed them to re-direct employees to work forms that better fit the current circumstances. Having flexibility meant managers could encourage employees to take breaks during the workday, approve time off work, reorganize work tasks, and spend extra time instructing the employee on the task at hand. Manager 13 described a situation where one of his employees was stressed and anxious and sick-listed part-time:

I tried to accommodate her work so that her work tasks changed in order for her to be able to contribute to the assignment while working from home and with a lesser work burden.

## Training and Education

Formalized training and education could be helpful. Managers thought it could improve their self-confidence, knowledge, and understanding of how to support employees and improve their skills in conflict management and communication. However, some managers had not experienced CMDs being discussed in any educational forum:

I don’t think I’ve ever been in a forum where that issue has been discussed. (Manager 31).

Handling cases of CMDs could be experienced as a situation whereby it was difficult to work according to the ideals that were taught in manager training; interactions related to CMDs required different skills. Training related to alcohol or drug abuse was described as providing clear guidelines about what was acceptable or not, whereas training regarding CMDs was experienced as more of a field of uncertainty, where managers were left to their own personal compassion when it came to what was acceptable behaviour or not:

Around drugs and alcohol, there is a clear ‘no–no’ line [included in manager training]. There is no grey zone or anything. But there is so much more around CMDs. It’s about your compassion, how you are, how you care about the employee. (Manager 31).

## Expert Functions

This category describes three organizational expert functions that were used either to provide the managers with knowledge of the employee’s situation or to support the managers directly. These functions were provided by the company or an external party.

### Human Resources

The human resources department (HR) could help managers to better understand CMDs in the company context. Managers mentioned that they often found mental health to be a frightening area to deal with; a resource that could provide support in better understanding the condition was valuable. HR had specific knowledge of CMDs and relevant laws and regulations, and they could explain the company culture and policy around CMDs. This was experienced as a safety net, especially for new managers who did not fully understand the organization’s way of working and the managerial role. They could also work as discussion partners when the managers needed validation of their reasoning and an outside view on the case. In a situation where an employee who had been absent without a physician’s certificate and the manager and employee were in a meeting together with HR, the manager felt relief when HR was being firm with the employee, reinforcing demands from an employer perspective. Being the one who made demands on a person with a CMD was experienced as uncomfortable, and in some cases, managers were afraid to ask questions out of fear of aggravating the mental state of the employee:

Since I myself wake up every morning and feel fine, I have a hard time understanding mental disorders. […] If a person feels really bad, you might even pose the wrong questions so that […] it might end with suicide, if I just go on in my usual way, as I do with persons who feel fine […] That is why you contact HR and try to get that help. (Manager 8).

Managers also described situations where HR did not have the knowledge on CMDs that they expected from an expert function, and they were left without any support at all. Good support from HR required some level of understanding of the situation. However, the HR department in some of the companies was in a different location than the manager. This was experienced as troublesome because HR then had poor insight into the local conditions of the company and might be completely unfamiliar with the employee’s circumstances, workplace, and work tasks. Manager 7 had discussed a situation with HR where an employee was close to fatigue depression but could not get a sick listing from his doctor and was unhappy with the response:

I meet these people every day, the HR people don’t. They can be really black and white while I see the whole person, I see how [CMDs] affect him.

### Occupational Healthcare Services Supporting the Employee

When OHS services supported employees with CMDs, it could be experienced as supportive for managers too, because it was a mental relief to leave the fears about how to handle CMDs to a professional party. OHS was given an authoritative role by some; they viewed the information provided by OHS as correct and neutral and attributed great importance to it when they assessed and organized the employee’s situation. By following the employee’s process in OHS, managers could increase their own knowledge, and sometimes OHS confirmed a situation that managers already suspected. The employee’s contact with OHS could result in new ways of communicating about mental health, which in turn made it easier for the manager and employee to understand each other. Manager 7 described how an employee had learnt to talk about mental health in terms of different colours of the days:

He can come to me and say, ‘Today it’s really dark’. ‘Okay, we’ll have to find you tasks that allow you to increase work little by little during the day, and we’ll speak again around lunch time to see if you feel more like light blue or if it’s going more towards black.’ This kind of tool has been great.

Support from OHS to the employee could also be problematic for managers because OHS was an external party and their insight into the specific workplace conditions was limited. Sometimes the communication from OHS was incomplete, gave rise to misunderstandings, and was too stretched out in time. The manager saw the case of CMD from an employer perspective, but OHS had more of a holistic view of the employee’s situation, which could lead to recommendations that were unreasonable from the managers’ point of view, resulting in feeling left alone in supporting the employee. In a situation where manager 15 needed support in planning work for an employee who was sick-listed part-time due to depression, the manager chose not to contact OHS:

They don’t have specific knowledge about our workplace… there is really no point in taking advice from someone who doesn’t understand the setting, the case, the limitations, and so on. If someone is limited in terms of capabilities to carry out one’s job, then there is no point in asking someone who doesn’t have any insight into the job.

### OHS Supporting the Manager

In situations where managers handled a case of an employee with a CMD, they sometimes received direct managerial support from the OHS, because these cases could be consuming and take up a lot of mental energy. Counselling with an OHS psychologist could help them to set up boundaries around their managerial role and to distinguish between being the employee’s manager and being their private self so that they would not be consumed by their commitment to the employee in their time off work. In other situations, the employee’s physician could have separate conversations with the manager, advising on what to consider in the situation from a manager’s perspective. Having someone to talk to was seen as a relief; many managers carried with them a sense of being alone with a heavy burden. In a situation where manager 11 experienced that the burden was becoming too heavy, she asked for counselling support, which turned out to make a big difference for her:

This case weighed really heavy on me, so I got in touch with a psychologist [through work]. It was really good for me, I learnt how to see [the case] in a different light. Before that, I carried it with me all the time, I took it home and some nights I hardly slept at all, and I felt really bad for the employee who was lonely.

## Safety Representative

Involvement of the safety representative (SR) in cases of CMD could be experienced as a guarantee that the manager was acting correctly in their employer role. This could provide managers with a sense of security in an otherwise unconfident situation because they trusted the SR to point out if they made mistakes and remind them about the measures they needed to take in certain situations. In a situation where manager 12 had to handle a case of CMD despite having very limited experience, she said:

[Involvement of SR] has made me think in new ways, that I have to offer support quicker. So it’s really good they’ve been here. It has opened my eyes more than in the past. SRs have not been around at our workplace like that before.

In meetings where the manager, the employee, and SR were present, managers could find it challenging to know how authoritative they could be towards the employee. Managers described situations where the SR could be tougher with the employee and speak up in ways that managers themselves were not comfortable with. When the SR explained the employee’s rights and obligations and clarified the importance of showing up at a meeting, the case became easier for the manager to handle because they did not have to take such an authoritative role. Managers who feared the risk of misunderstandings in meetings with the employee described that it gave them peace of mind to know that the SR also heard what was said in meetings.

In other situations, the SR could be passive, absent, or unnecessarily confrontational, as experienced by manager 30:

They take more of the employee’s perspective. And they really should. But it’s definitely not supportive from a manager’s perspective.

## Social Support from Management and Colleagues

This category describes four resources of support from management on different organizational levels.

### The Senior Management Team

The senior management team was supportive of managers when there was a consensus in the team on the values they wanted for the company, and they actively worked to communicate these values to the whole organization. Managers experienced that having shared values around mental health issues enabled them to have a close dialogue with senior management about how to handle cases of CMD and how to support employees. In a situation where manager 23 needed to accommodate work due to an employee’s mental health, the manager described that the consensus in the company’s small management team made it easy to find solutions for employees:

We have a very tight dialogue around each employee. […] It’s been really easy for us because we reason along the same lines when it comes to the work environment; it goes hand in hand with everything else. Employees are people and you must consider their personal situation, what the diagnosis is and so forth.

When the company’s senior management took an active interest in issues related to mental health, it also contributed to the work climate with a shared language around CMDs. This meant that managers did not have to exert themselves to bring mental health to the agenda and that mental distress could be discussed in the same way as any other topic in the organization.

Some managers experienced a lack of knowledge in their senior management team and asked for more commitment to issues of mental health. Manager 28, who experienced that she was the only one in the organization who had any sort of knowledge on CMDs (due to herself being burnt out some years ago), was frustrated that the team did not make a joint effort to learn more:

My suggestion is that we hire someone to educate us in the management team, so that we get updated, all of us. It can’t be that I should be the only one [with knowledge], every manager needs to have this knowledge and be able to see signals when something is not right with an employee.

### One’s Own Manager

Having an active dialogue with their senior manager could clarify the organizational frames that the managers had to work within and the possibilities available in the organization. It was experienced as critical that their own manager allowed them to take charge of the case in their own way. Involving one’s own manager meant a sense of security in that the senior managerial level was informed so that the managers were not left alone with the fear of being blamed for something that might go wrong in the handling of the case. Manager 4 explained that he had recurring check-up meetings with his own manager regarding an ongoing case of CMD:

I have this principle that I keep my senior manager informed about basically everything because it’s so much responsibility. I don’t want to be standing there alone if something were to happen.

However, the involvement of their own manager could be problematic when they did not share a view of the case or lacked knowledge about CMDs. To function as a resource, the senior manager needed to have some insight into the actual case. Sometimes managers were instructed to take actions that went against their own analyses of the case and what would be best for the employees; their managers often had a more rigid view of the situation. In a situation where manager 30 handled a case of fatigue depression case, she needed a temporary substitute from another part of the organization to take the employee’s place. Her request was not met with any understanding from her own manager:

I feel a little restricted. If my manager or my management team don’t have the same picture as I do and can support me in that, then I find it difficult to move forward. Then I get a little stuck and get very angry and frustrated as a manager, and I don’t get anywhere.

### Managerial Colleagues

Managerial colleagues at the same level in the organization could provide the managers with advice in the handling of specific cases. Conversations with colleagues gave them access to organizational knowledge that they were previously unaware of, and they could discuss cases, test ideas, and share experiences with each other. Managers could then feel safer and less alone and insecure in their decisions. Getting insights into how situations with CMDs had been handled previously by managerial colleagues made it easier for managers to support their employees. In a situation where manager 20 found herself struggling to handle her feelings about the impact her decisions could have on an employee with a CMD, she said:

When I’m the manager I in some ways influence other people’s lives… if I let someone go or force someone into rehabilitation and sick listing or so, then I influence their income, and it doesn’t always feel great to do that. I get a bad conscious from time to time… and I find the support we have in our managerial group, and from our senior manager, to be really good.

Where organizational support and knowledge were missing, managers experienced that their colleagues collectively avoided issues related to CMDs, as opposed to, for example, when an employee died. Manager 17 explained:

If someone dies, then everyone [of the managerial colleagues] approaches you to support and help. But if someone struggles a bit with their mental health, that’s something we don’t dare to handle. We should have more confidence there. Many shy away from these issues.

### Team Leaders

When managers had a subordinate team leader who supervised the employee’s work on a daily basis, the team leader could provide the manager with information about the employee’s health and capacity to work. The team leader had more daily knowledge about the employee and could observe the person at work, and was therefore able to give practical support to the manager. This made managers less alone in handling the case because they could divide the support to the employee between them. Manager 16 described a situation where an employee was on sick-leave due to exhaustion. To plan how to accommodate work, he was helped by information provided by a team leader, who oversaw the employee daily. He was insecure about which tasks were too challenging for the employee and thought that the employee herself probably could not decide that:

The team leader knows their job much better than I do as CEO, I’m probably too far away [from the operational work]. So they contribute with knowledge of the actual job, and they’re someone who can see that certain tasks are difficult. The team leader knows what tasks this employee shouldn’t do when she comes back.

## Interaction with Employees

This category describes three resources for good collaboration and communication with employees.

### Social Climate at the Workplace

An open climate where workplace knowledge about CMDs was prevalent could de-dramatize managers’ support to employees with CMDs. It helped the managers to check in with those who were having a hard time and to find ways to decrease their workload. Some managers described workplaces where conversations about mental health were ongoing and where employees opened up to managers if they struggled mentally. In a situation where manager 25 handled two cases, she explained that the trustful climate at their workplace had meant a lot for her abilities to support the employees:

[We want to] create that climate where you actually can find out what’s going on with the employees, how they really feel, not just showing up at meetings saying, ‘It’s a lot right now but it will probably have blown over in a week or so’, even though they think something else to themselves.

Some managers experienced difficulties talking about CMDs. A manager at a male-dominated workplace emphasized that the conversation climate was tough and characterized by “locker-room banter,” (Manager 7) which made work-related discussions on CMDs challenging. At some companies, time to discuss health issues was simply not a priority. The managers wished for a more open climate at the workplace, where everyone talked openly about their mental health and showed interest in their colleagues’ situation. They wished that it was acceptable to show one’s current mental state, even if it was not entirely positive, without making others in the group uncomfortable.

### An Involved Employee Group

When managers were able to communicate transparently with the whole workgroup about an employee with a CMD, they did not have to feel alone with the information. It was described as burdensome to not be open about the employee’s situation in front of the group. In a situation where manager 12 handled a case of CMD where the employee’s work task was to drive trucks, another employee from the group took extra note of the employee with CMD and supported the person in their daily work: “One of his colleagues offered to look after him and drive with him.” This meant a reduced mental load for the manager and a feeling of security.

Managers who did not sense any involvement in cases of CMD from their group wished they had been able to at least have a dialogue with a close colleague about the employee with CMD, to better understand the employee’s situation. Without that kind of information, they had a hard time understanding what the employee was going through and were not confident enough to communicate advice and support out of fear of doing wrong. Manager 6 said:

I’m sure it would’ve helped me to have a dialogue with a close colleague of hers. To get that understanding of what friends around her think, that could’ve helped.

### Physical Proximity to Employees

Having one’s office in the same building as employees made it possible to overview work and easily communicate with employees if needed because managers could be present to identify employees who seemed to struggle due to CMDs. Having physical proximity to employees and seeing them in everyday work gave managers a sense of their health state. Manager 33 described a situation where one employee had a history of being absent from work for periods, and the manager paid attention to her appearances:

I am in charge of this senior home, and I have my office right beside their [the employees’] computer so I see them really well. So I just asked [the employee with CMD] to step in to me when this sickness absence started again. […] I see every employee when they arrive, and [their mental state] shows quite clearly. I don’t manage that many employees, so I can keep track of them. If you catch them before [the CMDs get more severe], it doesn’t have to develop into long-term sickness absence.

Not having this closeness, however, made it more difficult to discover and understand employees’ troubles. Especially during the COVID-19 pandemic, managers experienced losing a sense of their employees’ mental state, because distance work made it more difficult to pick up signals and to initiate difficult conversations.

## Secondary Findings

During the analysis, we found that our organizational focus did not encompass every resource that the managers described; they included extra-organizational resources as well as their own personal resources in their accounts. When faced with an employee with a CMD, managers reached for all resources available to them, and particularly when organizational resources were insufficient.

External resources were directly or indirectly tied to the managers’ organization and were often routinely used in their support for employees with CMDs. These included authorities such as primary healthcare, the employment agency, the social insurance agency, the managers’ union organizations, and the employer organization to which their company was tied. These resources provided the managers with knowledge about CMDs, relevant legislation, boundaries for their employer responsibility, confirmation in their reasoning, and practical and emotional support in specific cases; something that they could not always get from their own company. In a situation where manager 27 did not know how to handle an employment related to CMDs, she needed to discuss the case with HR professionals. Her company did not provide an HR function, and she instead reached out to the employer organization and received help.

Personal resources such as the personal approach to leadership and experiences from private life were used to communicate with and understand the employee. Managers’ personal approach to leadership was a resource that helped them form their relationship with the employee by trusting their “gut feeling” in decision-making around the employee, committing emotionally to the case, taking responsibility to learn more about CMDs outside their office hours, and keeping an open mind. Manager 9 said:

I think it’s important that you keep yourself updated on these issues [CMDs]. You need to take responsibility for educating yourself.

Other managers described how they used their private time to read about CMDs and rehabilitation. There were also managers who had themselves had a CMD. Manager 5 explained how this helped him to notice changes in his employee’s behaviour: “I can spot the signals immediately because I’ve had them myself.”

## Discussion

This study explored the organizational resources that managers use when handling employees with CMDs and how they experience their use. Seven types of organizational resources, expressed as themes and sub-themes, were identified (Table 2). We also identified external and personal resources important for managers’ handling of employees with CMDs, presented as secondary findings. This is one of the first studies to explore managers’ real-life experiences of the support available to them in managing employees with CMDs. The findings acknowledge the importance of the context in which leadership occurs, which has often been overlooked in studies [50].

Our findings about managers’ experiences of organizational preconditions can be viewed through Johns’ framework of organizational context [4]. Certain organizational preconditions constrain the managers’ opportunities to support their employees, while others contain opportunities and function as organizational resources – for example, flexibility in work approaches and supportive managerial and collegial networks at different organizational levels. Johns’ framework distinguishes between discrete, meso och omnibus levels of context, each influencing organizational behaviour. At the discrete level, our findings identified specific job-related factors such as managers’ workload, autonomy, and collegial dynamics, all of which shape managers’ handling of employees with CMDs. At the meso level, this study focuses on the private sector, where profitability is a defining organizational characteristic. In this regard, one finding is that the senior managements’ engagement in mental health issues across the company was identified as an important supportive precondition. Additionally, managers sometimes experienced cultural constraints related to a male-dominated organizational demography in the workplace. Finally, although our study was not directed at the omnibus level, we observed the influence of broader contextual factors, such as national labour policies, which set overarching boundaries for managerial work. Among the findings, it was described that company routines and structures gave the managers support in adhering to such national regulations. Further, the labour unions’ organization is regulated at national level, and workplace safety representatives were experienced as influencing managers’ support to employees with CMDs. Our findings suggest that managers operate within and navigate preconditions on these levels simultaneously, with their ability to support employees depending on how these preconditions manifest across various layers of the organizational context.

The resources for *Routines and structures*, *Frames for action*, and *Expert functions* indicate that participating managers appreciated resources that allowed a balance between following specific guidelines and flexible use of managers’ own scope of action. For example, some managers in this study required clear guidance in terms of specific actions in individual cases, a preference that has been expressed in previous studies with managers [51]; other managers preferred flexible support allowing them greater autonomy in decision-making. A pattern emerged showing a kind of pendulum motion; when managers experience too much control from the organization, they may deviate from policy and make decisions they consider suitable despite going against the rules. The participating managers experienced that each employee with a CMD had different needs, and no general routine could be followed in every case. Sometimes managers turned to expert resources for the support needed in the specific case. Flexible and responsive expert functions seemed to be preferred by managers in this study because their support could then be adapted to the specific needs of the employee.

Uncertainty and frustration experienced by managers when organizational support is missing was a pattern across our findings. Our resource, *Social support from colleagues and management*, described managers’ experience of being the only one in the organization who had knowledge of CMDs. Experiences of a lack of support from one’s own manager were described, as well as avoidance from managerial colleagues. In previous interview studies, managers described experiencing a lack of support when handling employees with CMDs [12] and difficulties finding appropriate levels of support [52]. It has also been shown that it can be difficult for managers to assess their own responsibility, especially when problems appear to be related to private life situations [12], and they may feel that they are “groping in the dark” when working out strategies and solutions for the employee [13]. Together with our findings, these studies highlight the importance of providing managers with structures for collegiate and managerial support, so that they can develop confidence in their supporting role without being left to handle cases of CMD on their own.

When managers in our study were supported by their organization’s resources, they expressed feelings of relief and validation. It has previously been shown that managers’ emotions, especially self-confidence [53], can play a role in their support of employees. Suter et al. [52] interviewed managers at small companies and identified tensions in managing employees with CMDs, which included emotional demands and struggles. Limited knowledge and understanding of mental health issues and confidence in supportive actions have been described in earlier studies [1, 11, 38, 53]. This suggests that organizational resources can support CMD-related self-confidence in managers, for example, by providing organizational arenas for communication and learning about CMDs.

*Training and education* were identified as important in this study, described by managers to increase their knowledge, understanding, and confidence in CMD matters. However, experiences of insufficient or no managerial education focused on CMDs were also described. Managers have previously expressed that they lack the knowledge and competence needed to support employees adequately [1, 13]. This is noteworthy because previous cross-sectional studies have found associations between managers’ experience of training and how they accommodate and take proactive actions related to CMDs [37, 38]. A recent review of ten workplace interventions suggested that managerial training and education can increase health knowledge and improve non-stigmatizing attitudes and managers’ self-reported behaviour in supporting employees with CMDs [54]. However, behaviour is not driven simply by knowledge and information [55], and just raising awareness of mental health issues does not seem to encourage managers’ health-promoting behaviour [22]. Education aiming at changes in behaviour and action should be seen in their environmental and social context [56]; for education directed at managers, the logic of their organization needs to be taken into account. Training and education on CMDs might also need to be combined with clarification of managers’ organizational roles and responsibilities and what the benefits are for work-related outcomes [22]. Moreover, managerial training might need to be developed with an awareness of the different attitudes of male and female managers to mental health issues [30, 31].

Some barriers reduced managers’ possibilities to act with the help of organizational resources. Managers felt hindered in their support to employees, for example, when guidelines and structures did not allow for any flexibility in relation to the employee’s needs, or when expert functions in the organization lacked knowledge of workplace-specific conditions. Organizational knowledge gaps and stigma around CMDs could also be reasons why available resources are not used, as shown in our resources on *Social support from colleagues and management* and *Interaction with employees*. Previous qualitative research has indicated that organizational norms mediated by senior management may prevent managers from working with mental health promotion [22], suggesting that senior management plays an important role in establishing a supportive social climate around CMDs [3]. Contextual stigma at different organizational levels has been associated with fewer possibilities for managers to take action related to employees’ CMDs [33], and our resource, *Interaction with employees*, described that social climate could make workplace talk about CMDs impossible; ‘locker-room banter’ at the workplace made it difficult for the manager to communicate around mental health issues. This is in line with Eyllon et al. [57] who described that the masculine culture among construction workers contributed to stigma around mental health and perceptions of mental health as a female issue. Especially for managers in male-dominated workplaces, stigma can prevent the use of social resources. Hence, organizations need to work on establishing norms around mental health at all organizational levels and be attentive to the gender context of the organization.

The managerial level is also a layer of contextual stigma and includes contexts where managers interact with their colleagues. In the present study, *Social support from colleagues and management* was experienced as helpful when there was an open communication climate in collegial settings. Without the presence of stigma, managers experienced that they could speak freely about CMDs in collegial and organizational contexts and discuss how cases should be handled with regard to company norms and values. A recent cross-sectional study found negative associations between stigma at the collegial layer in organizations and managers’ possibilities for action [57], indicating that a stigma-free social context of managerial colleagues is an important resource. Stigma at managerial and organizational levels may reduce managers’ actions directed at CMDs [33], which is why positive organizational norms around mental health must be developed [22].

Our findings related to *Social support from colleagues and management* further showed that interaction with managerial colleagues enabled discussions around responsibility and actions, exchange of experiences, and learning organizational routines. Having a shared view of mental illness in the organization facilitated managers’ understanding and actions related to CMDs, which is in line with previous qualitative research indicating that senior management’s communication and engagement around health-related issues have an impact on managers’ sense of responsibility and their health-related leadership behaviour [22]. Organizations can provide support at the managerial collegial level in the form of peer support groups; managers have reported that they find it valuable to meet in groups and interact with mental health issues [51]. Collegial groups can strengthen managers’ confidence, and a supportive work environment for managers, where they are comfortable offering help to their employees, helps managers facilitate work performance and participation among employees with CMDs [58].

In line with Johns’ framework, our findings reflect managers’ experiences of situational opportunities and constraints [4]. An important finding is that the same organizational precondition could be experienced and described as both supportive and insufficient, reinforcing the argument that managers’ room for action is shaped by a variety of contextual factors. Our findings can be used to better tailor organizational resources and thereby better support managers.

## Methodological Considerations

The aim of this study was to explore organizational resources through the lens of managers’ experiences. Our data showed that managers used resources in a broader sense than just the organizational resources. We did not exclude these resources from the analysis even though they were strictly viewed as outside the aim. By including managers’ full experience of using external and personal resources as secondary findings, we were faithful to the participants’ real-life descriptions.

A strength of the study is the fact that the setting in terms of company profitability and private ownership was shared by all participants. Our findings, spanning a large variation in company size, suggest that managers need supportive organizational context no matter the size of the company. However, the private setting may limit transferability to public, tax-funded organizations.

For most managers, having an employee with CMD is a rare event. In this study, we defined a critical incident as a situation when a manager needed to support an employee with a CMD*.* It can be assumed that the experiences mentioned in the interviews concern situations that were challenging for the managers, introducing recall bias. The findings need to be interpreted as examples of situations that managers can experience. Quantitative studies are needed to establish the frequency of different types of management of employees with CMDs and other mental illnesses.

The trustworthiness of the study was established by having two interviewers calibrate their interviewing throughout the study. Further, the whole author group read the transcripts and identified the relevant meaning units for the data analysis.

The low number of participants from the pink sectors might reduce transferability to the pink sector where the main work object is human beings. However, although several studies show that employees with CMDs struggle most in interaction with other human beings [59–61], it is still likely that managers in the pink sector use and experience resources similar to the findings in this study.

Around one-third of the managers described a case involving alcohol abuse. In the interview, we specifically asked for cases involving depression, anxiety, or exhaustion. However, managers in general do not have thorough knowledge of the diagnostic system. As some managers described their cases, it became apparent that the CMDs were related to alcohol abuse, and managers sometimes expressed the alcohol abuse as self-medication of a CMD. We included these cases because we are interested in managers’ resources for support when they experience cases of CMD, even though the context of the disorders can vary.

The Swedish labour market system and welfare systems constitute part of the omnibus level of context [4] surrounding the organizational preconditions explored in this study. Some aspects of the welfare system are worth mentioning in relation to CMDs at workplaces, because they may have an impact on managers’ views on their contextual support and potentially limit the transferability of our findings to other social systems. In Sweden, employer responsibilities for the work environment and employee health are regulated by laws and universal social insurance systems. Swedish employers to some extent share the responsibility for employees with the national welfare system, which underlines the need for organizational support structures for managers. This may differ from other welfare contexts, where sick-leave might be paid for longer periods by the employer, or where more responsibility is placed on the employee through private insurances and welfare systems. In such situations, managers are likely to have different preconditions from which to handle CMDs at their workplace than the ones described in our study.

## Implications for Research and Practice

The present study can be used to gain deeper insight into managers’ experiences of their organizational prerequisites. These experiences emphasize that companies need to provide managers with adequate resources to enable them to act in cases of employees with CMDs. Organizations can use the findings of the study to review their own resources and identify how they can be developed. The findings can also be used to design survey questions to explore the issues on a larger scale.

Our findings suggest specific areas to consider when developing organizational support for managers, and that resources need to be adaptable to managers’ own needs and preferences. Social resources such as collegiate groups or communication with senior management were experienced by the participating managers as opportunities to discuss and understand matters related to CMDs. This can contribute to reducing stigmatization in the organizational context and the development of CMD-related organizational norms, which was identified as important for managers’ health-related behaviour in a review of qualitative studies [24]. Such groups also enable managers to pick up signals from senior management levels in the organization because senior management plays an important role in establishing top-down norms and standards that encourage managers to engage in mental health issues [24].

The findings can be used to design managerial education that contributes to increased self-confidence and knowledge. Martin et al. [62] found that managers use different types of knowledge when handling an employee case and developed a management education based on their findings. Their education included knowledge about what is typically done in situations involving CMDs, but also knowledge about how to execute the actions required in that situation. In our study, managers described situations where they felt their knowledge about CMDs was insufficient and where resources did not provide the guidance they needed. In line with Martin et al., our findings indicate that managerial education may include both knowledge about CMDs and knowledge about the consequences of CMDs in the context of the workplace and the actions they require.

Future research is needed to better establish the frequency of the use of the resources identified in this and other qualitative studies. Even if we recommend competence development regarding knowledge, attitudes, and managerial behaviour in supporting employees with CMD, we acknowledge that intervention studies are needed to establish the effectiveness of such measures. Knowledge is needed about the circumstances under which interventions may lead to changes in managerial behaviour and whether changes lead to improved support and management of employees with CMDs. Also, it is important to develop interventions directed at structural changes. As mentioned earlier, senior management has an important role in establishing norms, policies and transparent policies regarding roles and responsibilities in the organization. Structural changes should be evaluated for effects on managerial behaviour and outcomes related to the quality of employee support. Even if managers act according to best practices, employees might not experience helpful support. Thus, data on both manager and employee outcomes are needed.

This study concerned private companies with more than 50 employees. Future research should examine managers’ organizational prerequisites for support in public, tax-funded organizations and in smaller companies to enable comparisons of managerial resources across different organizational contexts. Given variations in organizational size and sector, managerial prerequisites likely differ [63, 64], particularly in terms of the flexibility for situational decision-making, which was found to be beneficial in the present study. Larger organizations may have more widespread knowledge of CMDs, whereas smaller private companies may allow for quicker decision-making. Thus, it would be valuable to investigate whether managers’ use of resources differs and whether they perceive resources as available and effective across different organizational settings.

## Conclusions

This study identifies seven types of organizational resources that managers experienced when supporting employees with CMDs, including routines and structures in the organization, frames for action, training and education, expert functions, safety representatives, managers and colleagues, and the employee group. Personal and external resources are presented as secondary findings. Managers’ experiences of these resources suggest ways for organizations to design resources to provide structure for the management of individual cases of CMDs at work. Resources flexible to the needs of managers in specific situations were experienced as supportive by participants in this study.

## Data Availability

Data supporting the findings of this study are available from the corresponding author upon reasonable request.
